# 한국 임상간호사의 의료기관 환경표면 소독 실무지침 수행도에 영향을 미치는 요인: 횡단적 연구

**DOI:** 10.7586/jkbns.26.004

**Published:** 2026-05-27

**Authors:** Do Gyoung Lee, Mi Yang Jeon

**Affiliations:** 1 Gyeongsang National University Changwon Hospital, Changwon, Korea; 1 창원경상국립대학교병원; 2 College of Nursing, Sustainable Health Research Institute, Gyeongsang National University, Jinju, Korea; 2 경상국립대학교 간호대학·지속가능건강연구소

**Keywords:** Disinfection, Evidence-based practice, Nurses, Practice guideline, Work performance, 소독, 근거기반실무, 간호사, 실무지침, 수행

## Abstract

**Purpose:**

This study aimed to identify factors influencing nurses’ performance of environmental surface disinfection guidelines in healthcare settings, with a focus on the perceived importance of the guidelines, evidence-based practice (EBP) beliefs, and organizational readiness for EBP.

**Methods:**

A cross-sectional study was conducted among 135 nurses working at a tertiary hospital and a general hospital. Data were collected from September 8 to September 30, 2024.

**Results:**

Performance of environmental surface disinfection guidelines differed significantly according to workplace (t = 2.60, *p* = .011) and length of experience in the current department (F = 5.64, *p* = .001). Nurses working in tertiary hospitals showed higher performance than those working in general hospitals, and nurses with more than 5 years of experience in their current department showed higher performance than those with less experience. Multiple regression analysis revealed that the perceived importance of the guidelines was the strongest predictor of performance (β = .53, *p* < .001), followed by organizational readiness for EBP (β = .22, *p* = .002) and EBP beliefs (β = .18, *p* = .012). The model explained 58.0% of the variance in performance (F = 59.31, *p* < .001, R^2^ = .58).

**Conclusion:**

Nurses’ performance of environmental surface disinfection guidelines is influenced by both individual and organizational factors, particularly the perceived importance of the guidelines, EBP beliefs, and organizational readiness for EBP. Strategies to improve performance should therefore emphasize increasing nurses’ awareness of guideline importance, fostering positive EBP beliefs, and enhancing organizational readiness.

## 서론

### 1. 연구의 필요성

의료관련감염(healthcare-associated infection, HAI)은 환자가 입원 당시 존재하지 않았거나 보유하지 않았던 감염이 병원이나 의료 시설에 입원하는 동안 획득된 감염을 의미한다[1]. HAI는 입원환자의 약 5%∼10%에서 발생하는 것으로 추정되며, 요로감염, 혈류감염, 폐렴 순으로 발생 빈도가 높은 것으로 보고되고 있다[2]. HAI가 발생하면 환자의 재원 기간이 길어지고 의료비용이 증가할 뿐만 아니라 사망 위험 또한 높아질 수 있다[3]. Zhang 등[4]은 미국 45개 병원에서 HAI로 인해 발생하는 연간 비용을 928,690달러로 추정하였고, Stewart 등[5]은 영국의 대형종합병원과 대학병원에서 HAI로 인한 초과 입원 기간이 평균 7.8일에 이른다고 보고하였다.

HAI는 환자 간 또는 환자와 의료인 간 직접 접촉뿐만 아니라 오염된 의료기관 환경표면을 매개로 전파될 수 있으며, 의료기관 환경표면은 병원체의 주요 저장소(reservoir)로 작용한다[6]. 침대 난간, 침상 주변 테이블, 호출벨과 같은 다빈도 접촉 표면은 의료진과 환자의 반복적인 접촉으로 쉽게 오염되며, 일부 병원체는 이러한 표면에서 생물막을 형성하여 수주에서 길게는 수개월까지 생존할 수 있는 것으로 보고되었다[7]. 환경표면 소독이 적절히 수행되지 않을 경우, 오염된 표면을 접촉한 의료진의 손이나 의료기기를 통해 병원체가 환자에게 전달되어 HAI 발생 위험을 증가시킬 수 있다[8]. 특히 입원 기간이 길어질수록 환자는 오염된 환경표면에 노출되는 시간이 증가하게 되며, 이는 환경표면을 매개로 한 HAI 위험을 높이는 주요 요인이 된다[9]. 최근 HAI를 예방하기 위해 의료기관 환경표면 관리와 소독의 중요성이 강조되고 있다.

의료기관 환경표면 관리는 물과 기계적 마찰, 세제를 이용하여 유기물과 이물질을 제거하는 청소(cleaning)와, 소독제를 사용하여 세균의 아포를 제외한 미생물을 제거하는 소독(disinfection)으로 구성된다[10]. 국외에서는 Centers for Disease Control and Prevention (2023) [11], Public Health Agency of Canada (2013) [12], Asia Pacific Society of Infection Control (2024) [13], Provincial Infectious Diseases Advisory Committee (2025) [14], National Health Service (2025) [15] 등에서 환경표면 소독 표준 지침을 제시하였다. 국내에서는 Korea Disease Control and Prevention Agency (KDCA, 2017) [16]에서 HAI 표준예방지침에 환경관리 지침을 포함하여 발표하였다. 그러나 코로나바이러스감염증-19 팬데믹 이후 환경표면 소독의 중요성이 더욱 부각됨에 따라 KDCA [10]에서 의료기관 환경표면 청소 및 소독 권고안을 새롭게 개발하였다. 이 지침에는 의료기관 환경표면 세척제와 소독제의 종류와 선택 기준, 세척제와 소독제의 희석 농도와 방법, 소독제의 접촉시간, 소독제를 사용할 때 기타 고려 사항, 소독 장비와 물품, 소독 티슈, 소독의 일반적 원칙, 소독의 주기, 소독의 방향, 다빈도 접촉 표면 소독, 설사를 유발하는 미생물에 대한 환경표면 소독, 퇴원 병실 소독 방법 등 임상현장에서 간호사가 직접 수행해야 하는 구체적인 환경표면 소독 실무 권고안을 포함하고 있다.

의료기관 환경관리와 관련된 국외 선행연구에서 간호사는 의료기관 환경관리에서 자신의 역할을 인지하고 있으나[17], 환경관리에서 역할의 모호성, 간호사의 환경관리 수행에 대한 지원 부족[17]과, 간호사가 의료기관 환경표면 소독을 수행하는 데 필요한 시간 부족과 업무량 과다가 수행에 영향을 미치는 요인으로 보고되었다[18]. 그러나 의료기관 환경표면 소독과 관련된 국내 연구는 병동 환경소독 모니터링 실태[19], 의료기관 환경표면 청소 및 소독 실태[20], 다제내성균의 환경표면 오염도 및 소독 효과 평가[21] 등과 실태 파악이나 소독의 효과를 평가하는 데 초점을 두고 있어, 간호사의 환경표면 소독 실무지침 수행에 영향을 미치는 요인을 분석하는 연구가 필요하다.

근거기반실무(evidence-based practice; EBP)는 의료서비스 제공에 있어서 최상의 연구 근거와 간호사의 전문 지식, 환자의 선호도 및 가치를 통합하여 환자 간호에 대한 최선의 결정을 내리는 문제 해결 접근 방식을 의미한다[22]. EBP를 수행한다는 것은 과학적 근거의 검색 및 평가, 동료 또는 환자와의 근거 및 자료 공유, 성과 자료의 수집 및 평가, 실무 변화를 위한 근거 활용과 관련된 행동을 하는 것을 포함하며[23], 이는 임상실무자들의 행동 변화를 넘어 전체 건강관리시스템을 바꾸는 복잡한 과정이다[24]. 따라서 EBP 실행을 위해서는 전반적인 시스템을 고려하는 것이 중요하다. EBP 확산 및 촉진과 관련된 모델 중 Advancing Research and Clinical Practice through Close Collaboration (ARCC) model은 EBP 조직환경과 지원을 강조하는 모델이다. Melnyk 등[22]은 ARCC model에서 EBP를 수행하는 데 관련되는 주요 변수로 EBP에 대한 조직문화와 준비도, 지식, 신념과 역량을 제시하였으며, 조직문화와 준비도, 지식, 신념과 역량은 EBP 수행과 유의한 정적 상관관계가 있다고 설명된다.

선행연구에서 EBP 수행도에 간호사의 개인적 요인으로는 EBP 중요도 인식과 수행의 필요성에 대한 인식 수준[25,26]과 EBP 신념[25-27], EBP 지식[28]과 EBP 역량[29]이, 조직적 요인으로는 근거기반실무 수행을 지원하는 조직 문화와 조직준비도[24,27]가 영향을 미치는 요인으로 보고하였다. 그러나 현재까지는 EBP 수행에 영향을 미치는 요인을 개인적 요인과 조직적 요인으로 구분하여 분석되고 왔고, 개인적 요인과 조직적 요인을 통합적으로 분석한 연구는 거의 이루어지지 않았다. 특히 환경표면 소독 실무지침은 간호사뿐 아니라 여러 직종이 관여하는 영역이므로, 간호사의 개인적 요인과 의료기관의 조직적 요인이 상호작용할 수 있다. 따라서 이들 요인이 수행에 미치는 영향을 통합적으로 분석할 필요가 있다.

이에 본 연구는 임상간호사를 대상으로 의료기관 환경표면 소독 실무지침 수행도에 영향을 미치는 요인을 간호사의 개인적 요인인 실무지침 중요도 인식과 근거기반실무 신념과 조직적 요인인 근거기반실무 조직준비도를 중심으로 통합적으로 분석함으로써 의료기관 환경표면 소독 실무지침의 수행을 증진하기 위한 근거를 마련하고자 한다.

### 2. 연구의 목적

임상간호사의 의료기관 환경표면 소독 실무지침 수행도에 영향을 미치는 요인을 규명하고, 실무지침 수행도를 향상시키기 위한 전략을 개발하는 데 필요한 기초자료를 제공하고자 하며, 구체적인 목적은 다음과 같다.

1) 임상간호사의 일반적 특성, 직무 관련 특성, 의료기관 환경표면 소독 실무지침 수행도와 중요도, 근거기반실무 신념, 근거기반실무 조직준비도를 파악한다.

2) 임상간호사의 일반적 특성 및 직무 관련 특성에 따른 의료기관 환경표면 소독 실무지침 수행도의 차이를 확인한다.

3) 임상간호사의 의료기관 환경표면 소독 실무지침 수행도와 중요도, 근거기반실무 신념, 근거기반실무 조직준비도 간의 상관관계를 확인한다.

4) 임상간호사의 의료기관 환경표면 소독 실무지침 수행도에 영향을 미치는 요인을 규명한다.

## 연구 방법

### 1. 연구 설계

본 연구는 임상간호사의 의료기관 환경표면 소독 실무지침 수행도에 영향을 미치는 요인을 파악하기 위한 횡단적 서술적 상관관계 연구(cross-sectional descriptive correlational study)이다. 본 연구는 관찰연구 보고지침인 STROBE (Strengthening the Reporting of Observational Studies in Epidemiology) Statement를 참고하여 보고하였다.

### 2. 연구 대상

임상간호사를 대상으로 하였으며, 연구의 목적과 절차를 이해하고 자발적으로 연구 참여에 동의한 간호사를 연구 대상으로 하였다. 대상자 선정 기준에서 포함 기준은 현재 종합병원급 이상 의료기관에서 근무 중이며 임상 경력이 1년 이상인 간호사였다. 본 연구에서 임상경력 1년 이상인 간호사를 대상자로 선정한 근거는 간호사의 감염예방 지식과 수행은 근무 경력과 유의한 상관관계가 있으며[30], 임상경력 1년 미만인 신규간호사는 감염관리 지식은 높지만 시간적 압박, 숙련도 부족 등으로 인해 실제 수행 수준이 낮다[31]고 보고한 선행연구 결과이다. 이를 근거로 임상경력 1년 이상인 간호사가 의료기관 환경표면 소독 관련 간호업무를 독립적이고 안정적으로 수행할 것으로 판단하여 본 연구의 대상자로 선정하였다. 대상자 제외 기준은 의료기관 환경표면 소독을 직접 수행하지 않는 간호사로 하였다. 본 연구는 비확률적 편의표집(convenience sampling) 방법을 사용하여 대상자를 선정하였다.

표본 크기는 G*Power 3.1.9 프로그램을 이용하여 다중회귀분석에 필요한 표본 수를 산출하였다. 효과크기 .15(중간 효과크기), 유의수준 .05, 검정력 .80, 독립변수 11개를 기준으로 산출한 최소 표본 수는 123명이었다. 탈락률 약 20%를 고려하여 총 148명에게 설문지를 배부하였다. 설문조사 결과, 불성실하게 응답한 대상자 13명을 제외하고, 총 135명을 최종 분석 대상자로 선정하였다.

### 3. 연구 도구

#### 1) 대상자의 일반적 특성

본 연구에서 대상자의 일반적 특성은 성별, 연령, 학력을 포함한 총 3개 문항으로 구성하였다. 직무 관련 특성은 의료기관 환경표면 소독 실무지침 수행과 관련성이 있을 것으로 판단되는 변수로서, 총 임상경력, 근무기관 종류, 현 근무부서, 현 부서 근무경력, 최근 1년 이내 감염관리 교육 경험 유무를 포함한 총 5개 문항으로 구성하였다.

#### 2) 의료기관 환경표면 소독 실무지침 수행도

의료기관 환경표면 소독 실무지침 수행도는 KDCA [11]에서 제시한 의료기관 환경표면 청소 및 소독 권고안에 포함된 총 76개의 환경표면 소독 권고안을 근거로, 본 연구팀이 개발한 수행도 측정 도구를 사용하여 측정하였다. 도구 개발 과정에서 중복되는 권고안 5개와 간호사의 실무와 직접적인 관련성이 낮은 권고안 4개를 제외하여 총 67개 문항을 1차 도구의 문항으로 선정하였다.

도구의 내용타당도 검증은 권고안 개발자 2인과 감염관리 분야 전문가 2인을 포함한 총 4인의 전문가를 대상으로 2차에 걸쳐 실시하였다. 1차 전문가 내용타당도를 검증한 결과, 문항별 내용타당도 지수(Item-level Content Validity Index, I-CVI)가 0.50∼0.75에 해당하는 문항 20개를 삭제하였다. 이후 수정된 47개 문항에 대해 2차 전문가 내용타당도 검증을 실시한 결과, 모든 문항의 I-CVI는 0.80∼1.00 범위로 나타났으며, 추가적인 문항 수정 의견이 없어 47개 문항으로 최종 도구를 확정하였다. 이 도구는 총 12개 하위영역, 47개 문항으로 구성되었으며, 하위영역별 문항 수는 ‘세척제와 소독제의 종류와 선택 기준’ 4개 문항, ‘세척제와 소독제의 희석 농도와 방법’ 2개 문항, ‘소독제의 접촉시간’ 1개 문항, ‘소독제를 사용할 때 기타 고려 사항’ 3개 문항, ‘소독 장비와 물품’ 5개 문항, ‘소독 티슈’ 3개 문항, ‘소독의 일반적 원칙’ 2개 문항, ‘소독의 주기’ 4개 문항, ‘소독의 방향’ 7개 문항, ‘다빈도 접촉 표면 소독’ 4개 문항, ‘설사를 유발하는 미생물에 대한 환경표면 소독’ 10개 문항, ‘퇴원 병실 소독 방법’ 2개 문항이다.

각 문항별 점수는 4점 Likert 척도로 구성되었으며, ‘전혀 수행하지 않는다’ 1점, ‘가끔 수행한다’ 2점, ‘자주 수행한다’ 3점, ‘항상 수행한다’ 4점으로 응답하도록 하였다. 점수가 높을수록 의료기관 환경표면 소독 실무지침 수행 수준이 높음을 의미한다. 본 연구에서 1개 문항으로 구성된 소독제의 접촉시간을 제외한 하위영역별 도구의 신뢰도 Cronbach’s α는 세척제와 소독제의 종류와 선택 기준 .66, 세척제와 소독제의 희석 농도와 방법 .84, 소독제를 사용할 때 기타 고려 사항 .62, 소독 장비와 물품 .73, 소독 티슈 .85, 소독의 일반적 원칙 .79, 소독의 주기 .83, 소독의 방향 .90, 다빈도 접촉 표면 소독 .87, 설사를 유발하는 미생물에 대한 소독 .93, 퇴원 병실 소독 방법 .82이며 전체 도구의 Cronbach’s α는 .96이었다.

#### 3) 의료기관 환경표면 소독 실무지침 중요도

의료기관 환경표면 소독 실무지침 중요도는 본 연구에서 개발한 의료기관 환경표면 소독 실무지침 수행도 도구의 문항을 기반으로 개발하였다. 본 도구는 환경표면 소독 실무지침 각 항목에 대해 간호사가 인식하는 중요도를 측정하기 위한 것으로, 수행도 도구와 동일하게 총 12개 하위영역, 47개 문항으로 구성되었다. 각 문항별 점수는 4점 Likert 척도이며, ‘전혀 중요하지 않다’ 1점, ‘중요하지 않다’ 2점, ‘중요하다’ 3점, ‘매우 중요하다’ 4점으로 구성되었으며, 점수가 높을수록 의료기관 환경표면 소독 실무지침에 대한 중요성 인식 수준이 높음을 의미한다. 수행도 도구와 동일한 문항 구조를 사용함으로써 수행 수준과 중요도 인식 간의 비교 분석이 가능하도록 설계하였다. 본 연구에서 1개 문항으로 구성된 소독제의 접촉시간을 제외한 하위영역별 도구의 신뢰도 Cronbach’s α는 세척제와 소독제의 종류와 선택 기준 .83, 세척제와 소독제의 희석 농도와 방법 .87, 소독제를 사용할 때 기타 고려 사항 .77, 소독 장비와 물품 .86, 소독 티슈 .86, 소독의 일반적 원칙 .82, 소독의 주기 .87, 소독의 방향 .93, 다빈도 접촉 표면 소독 .82, 설사를 유발하는 미생물에 대한소독 .89, 퇴원 병실 소독 방법 .96이며 도구 전체의 신뢰도 Cronbach’s α는 .98이었다.

#### 4) 근거기반실무 신념

본 연구에서 근거기반실무 신념은 Melnyk 등[23]이 개발한 Evidence-based practice beliefs scale을 Park과 Jang [32]이 한국어로 번역한 도구를 사용하여 측정하였으며, 도구 개발자로부터 사용 승인을 받은 후 사용하였다. 본 도구는 간호사가 근거기반실무의 가치와 유용성, 근거기반실무 수행에 대한 자신감과 긍정적 태도를 얼마나 가지고 있는지를 측정하기 위한 도구이다.

이 도구는 총 16개 문항으로 구성되어 있으며, 5점 Likert 척도를 사용하였다. 각 문항의 점수는 ‘매우 부정적이다’ 1점, ‘부정적이다’ 2점, ‘보통이다’ 3점, ‘긍정적이다’ 4점, ‘매우 긍정적이다’ 5점으로 구성되어 있고, 점수가 높을수록 근거기반실무 신념 수준이 높음을 의미한다. 문항 중 11번과 13번은 부정 문항으로, 분석 시 역환산하여 합산하였다. 도구 개발 당시 Melnyk 등[23]의 연구에서 Cronbach’s α는 .87이었으며, Park과 Jang [32]의 연구에서는 Cronbach’s α가 .85로 보고되었다. 본 연구에서 근거기반실무 신념 도구의 신뢰도 Cronbach’s α는 .89였다.

#### 5) 근거기반실무 조직준비도

본 연구에서 근거기반실무 조직준비도는 Fineout-Overholt와 Melnyk [33]가 개발한 Organizational culture and readiness for system-wide implementation of evidence-based practice 도구를 Park과 Jang [32]이 한국어로 번역하고 수정한 도구를 사용하여 측정하였으며, 도구 개발자로부터 사용 승인을 받은 후 사용하였다. 이 도구는 조직 차원에서 근거기반실무를 수행할 수 있는 문화와 환경이 어느 정도 준비되어 있는지를 평가하기 위한 것으로, 조직의 리더십 지지, 자원과 인프라, 교육과 지원체계, 근거기반실무에 대한 조직 문화 등을 포함한다.

본 도구는 총 25개 문항으로 구성되어 있으며, 문항별 점수는 5점 Likert 척도를 사용하였다. 각 문항의 점수는 ‘전혀 없음’ 1점, ‘거의 없음’ 2점, ‘보통’ 3점, ‘대체로 잘 되어 있음’ 4점, ‘매우 잘 되어 있음’ 5점으로 구성되어 있고, 점수가 높을수록 근거기반실무에 대한 조직준비도가 높음을 의미한다. 도구 개발 당시 Fineout-Overholt와 Melnyk [33]의 연구에서 Cronbach’s α는 .85였으며, Park과 Jang [32]의 연구에서는 Cronbach’s α가 .95로 보고되었다. 본 연구에서 근거기반실무 조직준비도 도구의 신뢰도 Cronbach’s α는 .96이었다.

### 4. 자료 수집

본 연구는 진주시에 소재한 경상국립대학교 생명윤리위원회(Institutional Review Board, IRB)의 승인을 받은 후 2024년 9월 8일부터 2024년 9월 30일까지 자료를 수집하였다. 대상자 모집은 비확률적 편의표집을 기반으로 한 연쇄표집(convenience-based chain-referral sampling) 방법을 사용하였다.

자료 수집을 위해 특정 의료기관을 지정하지 않고, 종합병원급 이상 의료기관에 근무하는 간호사 중 연구자와 알고 있는 간호사 10명에게 전화로 연구의 목적과 절차를 설명한 후 연구의 목적과 참여 절차를 이해하고 연구 참여에 자발적으로 동의한 간호사에게 온라인 설문지 링크를 이메일 또는 문자메시지로 전달하였다. 최초로 설문에 참여한 간호사는 연구 참여를 희망하는 다른 간호사에게 연구 정보를 안내하였으며, 이후 연구 참여를 희망한 간호사가 자발적으로 자신의 연락처를 연구자에게 제공하면, 설문지 링크를 발송하였다. 온라인 설문은 Naver Form 서비스를 이용하여 조사하였다. 온라인 설문지는 설문 링크에 접속 시 첫 화면에서 ‘설문 참여에 동의한다’ 또는 ‘설문 참여에 동의하지 않는다’를 선택하도록 구성하였다. 연구 참여에 자발적으로 동의한 경우에만 설문 문항에 응답할 수 있도록 설계하였으며, 모집 인원이 충족되면 설문이 자동으로 종료되도록 설정하여 추가 응답이 불가능하도록 하였다.

### 5. 자료 분석

본 연구에서 수집된 자료는 SPSS version 24.0 (IBM Corp., Armonk, NY, USA)으로 분석하였으며 구체적인 방법은 다음과 같다.

1) 대상자의 일반적 특성과 직무 관련 특성은 기술통계를 이용하여 빈도와 백분율, 평균과 표준편차로 분석하였다.

2) 의료기관 환경표면 소독 실무지침 수행도와 중요도, 근거기반실무 신념, 근거기반실무 조직준비도는 평균과 표준편차로 분석하였다.

3) 대상자의 일반적 특성과 직무 관련 특성에 따른 의료기관 환경표면 소독 실무지침 수행도의 차이는 정규성 검증 결과에 따라 분석하였다. 정규성 검증은 Shapiro–Wilk test를 이용하였다. 정규분포를 가정할 수 있는 경우 독립표본 t-검정(independent t-test)과 일원배치분산분석(one-way analysis of variance)를 실시하였으며, 사후검정은 Scheffé test를 이용하였다. 정규분포를 가정할 수 없는 경우에는 Mann–Whitney U test와 Kruskal–Wallis test를 실시하였고, Kruskal–Wallis test에서 유의한 차이가 있는 경우 Mann–Whitney U test를 이용하여 사후검정을 실시하였으며, 이때 Bonferroni correction을 적용하여 유의수준을 보정하였다.

4) 의료기관 환경표면 소독 실무지침 수행도, 중요도, 근거기반실무 신념, 근거기반실무 조직준비도 간의 상관관계는 Pearson의 상관계수(Pearson correlation coefficient)를 이용하여 분석하였다.

5) 대상자의 의료기관 환경표면 소독 실무지침 수행도에 영향을 미치는 요인은 단계적(Stepwise) 선택 방식의 다중회귀분석(multiple linear regression analysis)으로 분석하였다. 변수 선택은 유의확률(*p*-value)을 기준으로 하였으며, 변수 투입은 .05 이하, 변수 제거는 .10 이상으로 설정하였다. 회귀분석에 앞서 오차항의 독립성, 정규성, 등분산성을 검토하였으며, 독립변수 간 다중공선성 여부는 공차한계(tolerance)와 분산팽창요인(variance inflation factor, VIF)을 통해 확인하였다.

6) 연구 도구의 내적 일관성 신뢰도는 Cronbach’s α 계수를 이용하여 분석하였다.

### 6. 윤리적 고려

본 연구는 경상국립대학교 생명윤리위원회(IRB)의 심의를 거쳐 연구의 목적, 방법, 대상자 권리 보장 및 설문지 내용에 대해 승인을 받은 후 진행하였다(IRB 승인번호: GIRB-A24-NY-0089). 연구 대상자에게 연구의 목적과 절차, 자료 수집 방법을 충분히 설명하였으며, 연구 참여는 전적으로 자발적인 선택에 의해 이루어졌고, 참여 여부에 따라 어떠한 불이익도 없음을 명확히 고지하였다. 연구 참여에 자발적으로 동의한 대상자에게 사전 동의를 받은 후 설문조사를 시행하였다. 본 연구에서 수집된 자료는 비밀보장(confidentiality) 원칙에 따라 관리되었다. 설문 응답 자료에는 개인을 식별할 수 있는 정보가 포함되지 않았으며, 연구 참여에 대한 답례품 제공을 위해 수집된 연락처 정보는 설문 응답 자료와 분리하여 관리하였다. 모든 자료는 연구 목적 외에는 사용되지 않았고, 온라인 설문을 통해 수집된 자료는 비밀번호가 설정된 개인 컴퓨터에 저장되었으며, 연구 종료 후 관련 자료는 규정에 따라 파기할 것임을 대상자에게 설명하였다. 온라인 설문지는 설문 링크 접속 시 첫 화면에서 ‘설문 참여에 동의한다’ 또는 ‘설문 참여에 동의하지 않는다’를 선택하도록 구성하였으며, 연구 참여에 동의한 경우에만 설문 문항에 응답할 수 있도록 하였다. 설문 참여에 동의하지 않은 대상자의 경우, 어떠한 개인정보도 수집되지 않으며 즉시 종료되도록 하였다.

연구 참여에 대한 감사의 표시로 설문을 완료한 대상자에게 5,000원 상당의 모바일 기프티콘을 제공하였다. 답례품 제공을 위해 수집된 연락처 정보는 기프티콘 발송 후 즉시 파기하였으며, 답례품 제공은 연구 참여를 강제하거나 영향을 미치지 않도록 소정으로 제공되었다.

## 연구 결과

### 1. 대상자의 일반적 특성과 직무 관련 특성

본 연구 대상자의 일반적 특성에서 평균 연령은 30.37 ± 5.89세였으며, 연령군 중에서는 26∼30세가 76명(56.3%)으로 가장 높은 비중을 차지하였다. 성별은 여성이 128명(94.8%)으로 대부분을 차지하였고, 남성은 7명(5.2%)이었다. 교육 수준은 4년제 졸업이 93명(68.9%)으로 가장 많았으며, 석사 재학 이상 34명(25.2%), 3년제 졸업 8명(5.9%) 순으로 나타났다(Table 1).

직무 관련 특성에서 대상자의 총 임상경력은 평균 6.94 ± 5.19년이었으며, 경력군 중에서는 5∼10년 미만이 68명(50.4%)으로 가장 큰 비중을 차지하였다. 근무기관은 상급종합병원 72명(53.3%), 종합병원 63명(46.7%)으로 나타났다. 근무부서는 일반병동 68명(50.4%)과 특수부서 67명(49.6%)로 유사한 분포를 보였으며, 특수부서에는 중환자실, 응급실, 수술실, 분만실, 신생아실이 포함되었다.

현 근무부서 경력은 평균 3.73 ± 4.07년이었으며, 경력 범주 중에서는 5년 이상이 35명(25.9%)으로 가장 높은 비중을 차지하였다. 감염관리 교육 경험이 있는 대상자는 125명(92.6%)이었고, 없는 경우는 10명(7.4%)이었다(Table 1).

### 2. 의료기관 환경표면 소독 실무지침 수행도와 중요도, 근거기반실무 신념, 근거기반실무 조직준비도의 점수

의료기관 환경표면 소독 실무지침 수행도 평균 평점은 4점 만점에 3.27 ± 0.49점이었다. 하위영역별로는 의료기관 환경표면에 사용되는 세척제와 소독제의 종류와 선택 기준(3.41 ± 0.48점), 설사를 유발하는 미생물에 대한 환경표면(3.36 ± 0.57점) 순으로 점수가 높았다. 반면, 의료기관 환경표면 소독제의 접촉시간(2.92 ± 0.99점)과 퇴원환자 병실 소독 방법(2.97 ± 0.95점) 순으로 점수가 낮았다. 의료기관 환경표면 소독 실무지침 하위영역의 수행도 평균 평점의 범위는 2.92∼3.41점으로 나타났다(Table 2).

의료기관 환경표면 소독 실무지침 중요도 평균 평점은 4점 만점에 3.66 ± 0.35점이었다. 하위영역별로는 의료기관 환경표면 소독제를 사용할 때 기타 고려 사항(3.72 ± 0.38점), 설사를 유발하는 미생물에 대한 환경표면(3.72 ± 0.37점), 다빈도 접촉 표면 소독(3.70 ± 0.40점) 순으로 점수가 높았다. 반면, 퇴원 병실 소독 방법(3.57 ± 0.53점)과 의료기관 환경 소독제의 접촉시간(3.57 ± 0.55점) 순으로 점수가 낮았다. 의료기관 환경표면 소독 실무지침 하위영역의 중요도 평균 평점의 범위는 3.57∼3.72점이었다.

근거기반실무 신념 평균 평점은 5점 만점에 3.75 ± 0.48점이었으며, 근거기반실무 조직준비도 평균 평점은 5점 만점에 3.19 ± 0.78점이었다(Table 2).

### 3. 일반적 특성과 직무 관련 특성에 따른 의료기관 환경표면 소독 실무지침 수행도의 차이

의료기관 환경표면 소독 실무지침 수행도는 연령, 성별, 학력에 따른 유의한 차이가 없었다. 직무 관련 특성 중에서는 근무기관(t = 2.60, *p* = .011)과 현 근무부서 경력(F = 5.64, *p* = .001)에 따라 수행도에 유의한 차이가 있었다. 상급종합병원에 근무하는 간호사는 종합병원에 근무하는 간호사보다 의료기관 환경표면 소독 실무지침 수행도가 유의하게 높았으며, 현 근무부서 경력 5년 이상인 집단은 1~3년 미만과 3~5년 미만 집단보다 수행도가 유의하게 높았다(Table 1).

### 4. 의료기관 환경표면 소독 실무지침 수행도와 의료기관 환경표면 소독 실무지침 중요도, 근거기반실무 신념, 근거기반실무 조직준비도간의 상관관계

의료기관 환경표면 소독 실무지침 수행도는 의료기관 환경표면 소독 실무지침 중요도(r = .68, *p* < .001), 근거기반실무 신념(r = .50, *p* < .001), 근거기반실무 조직준비도(r = .53, *p* < .001)와 모두 정적 상관관계를 보였다(Table 3).

### 5. 의료기관 환경표면 소독 실무지침 수행도에 영향을 미치는 요인

의료기관 환경표면 소독 실무지침 수행도에 영향을 미치는 요인을 규명하기 위해, 직무 관련 특성 중 수행도에 유의한 차이를 보인 근무기관(종합병원 기준)과 현 근무부서 경력(3~5년 미만 기준), 그리고 수행도와 유의한 상관관계를 보인 의료기관 환경표면 소독 실무지침 중요도, 근거기반실무 신념, 근거기반실무 조직준비도 총 5개의 변수를 독립변수로 투입하여 다중회귀분석을 실시하였다. 회귀분석에 앞서 오차항의 독립성, 정규성 및 등분산성을 검토한 결과, Durbin–Watson 통계량은 1.98로 오차항 간 자기상관이 없었으며, 표준화 잔차가 모두 ±3 이내로 나타나 극단적인 이상치는 확인되지 않았으며, 잔차의 정규성 가정을 크게 위반하지 않는 것으로 판단하였다. 또한 Breusch–Pagan 검정 결과, 유의확률이 .571로 .050 이상 높은 것으로 나타나 등분산성이 확인되었고, 다중공선성을 공차한계와 VIF로 검토한 결과, 공차한계는 .66∼.80으로 .10 이상이었으며, 분산팽창지수는 1.24∼1.50으로 10.00 이하의 값으로 나타나 독립변수 간 다중공선성은 없는 것으로 나타났다.

다중회귀분석 결과, 의료기관 환경표면 소독 실무지침 수행에 가장 큰 영향을 미치는 변수는 의료기관 환경표면 소독 실무지침 중요도(β = .53, *p* < .001)였으며, 다음으로 근거기반실무 조직준비도(β = .22, *p* = .002)와 근거기반실무 신념(β = .18, *p* = .012)이 유의한 영향을 미치는 것으로 나타났다. 즉, 의료기관 환경표면 소독 실무지침 중요도, 근거기반실무 조직준비도, 근거기반실무 신념이 높을수록 수행도 수준이 상대적으로 높게 나타났다. 본 회귀모형의 설명력은 58.0%였으며, 모형은 통계적으로 유의하였다(F = 59.31, *p* < .001, R^2^ = .576) (Table 4).

## 논의

본 연구는 임상간호사의 의료기관 환경표면 소독 실무지침 수행에 영향을 미치는 요인을 규명하고자 수행되었으며, 주요 연구 결과를 중심으로 논의하고자 한다. 본 연구에서 의료기관 환경표면 소독 실무지침 수행도는 4점 만점에 평균 3.27점으로 나타나 전반적으로 중등도 이상의 수행 수준을 보였다. 그러나 하위영역별 점수는 2.92점에서 3.41점의 범위를 나타내어 수행 수준 간 편차가 존재함을 확인할 수 있었다. 이러한 결과는 동일한 측정 도구를 사용하지는 않았으나 의료기관 환경표면 청소·소독 실무지침 수행도를 조사한 Jeong 등[20]의 연구에서 보고된 3.27∼3.83점과 비교할 때 전반적으로 낮은 수준으로 해석될 수 있다. 이는 본 연구가 의료기관 환경표면 ‘소독’ 실무지침 수행에 초점을 둔 반면, Jeong 등[20]의 연구는 ‘청소·소독’을 포함한 보다 포괄적인 개념을 다루었다는 점에서 지침의 범위와 내용의 차이에 기인했을 가능성이 있다.

한편, Al Hajjeh 등[34]이 아부다비 소재 일개 병원에서 보고한 환경 청소·소독 절차 준수율 45% 및 Ahmed, Soliman과 Mohamed [35]이 수술실 간호사를 대상으로 보고한 환경표면 소독 수행률 75%와 비교할 때, 본 연구 대상자의 수행 수준은 상대적으로 높은 것으로 나타났다. 이러한 차이는 국가 간 감염관리 정책, 의료기관 인증제도, 그리고 조직 차원의 감염관리 체계의 차이와 관련이 있을 가능성을 시사한다. 특히 대한민국의 경우 2021년부터 의료기관 인증평가 항목에 환자치료 영역의 환경관리 기준이 포함되면서 청소 및 소독 수행에 대한 관리·감독과 교육이 강화되었으며[36], 이러한 제도적 요인이 수행 수준 향상과 관련이 있을 가능성이 있다. 또한 코로나바이러스감염증-19 팬데믹을 경험하면서 환경표면 소독의 중요성에 대한 인식이 증대된 점 역시 수행도 향상에 기여했을 것으로 해석될 수 있다. 다만 이러한 해석은 횡단적 연구 설계의 특성상 인과관계보다는 관련성 수준에서 이해할 필요가 있다.

본 연구에서 특히 주목할 점은 환경표면 소독의 하위영역 중 퇴원환자 병실 소독방법과 소독제 접촉시간 준수 영역의 수행도가 상대적으로 낮게 나타났다는 점이다. 이는 간호사의 업무량 과다와 시간적 제약이 감염관리 지침 수행에 영향을 미칠 수 있음을 보고한 선행연구[18]와 일관된 결과이다. 즉, 환경표면 소독 중에서도 일정 시간 이상 소독제를 유지해야 하는 접촉시간 준수와 같은 시간 의존적 수행행위는 임상 현장에서의 업무 강도와 시간적 제한으로 인해 실제 수행이 제한될 가능성이 있음을 시사한다. 이는 간호사들이 의료기관 환경표면 소독 실무지침을 수행하는 과정에서 시간적 여유의 부족과 업무량 과다가 수행에 영향을 미칠 수 있음을 보고한 선행연구[18]의 결과와 맥락을 같이한다. 이러한 결과는 환경표면 소독 실무지침 수행이 간호사 개인의 단순한 지식이나 태도뿐 아니라 임상 환경에서의 구조적 제약과 업무 흐름과 밀접하게 관련된 행위임을 의미한다. 따라서 수행도를 향상시키기 위해서는 개별 간호사의 노력에만 의존하기보다는 조직 차원의 체계적 접근이 요구된다. 구체적으로, 환경표면 소독 절차에 대한 체크리스트를 개발하여 적용한다면 수행 과정에서의 인지적 부담을 감소시키고 절차 누락을 예방함으로써 지침 수행도를 향상시키는 데 기여할 것이다. 또한 환경표면 소독과 관련된 간호사의 업무 부담을 완화시키고, 업무 효율성을 향상시키기 위해 다빈도 접촉표면 관리에 대한 책임은 간호사가 유지하되, 일부 반복적이고 표준화 가능한 업무는 보조인력에게 위임하는 체계를 명확히 할 필요가 있다. 다만 이러한 업무 위임은 표준화된 수행 지침과 적절한 감독 체계를 기반으로 이루어져야 한다.

본 연구에서는 상급종합병원에 근무하는 간호사가 종합병원에 근무하는 간호사보다 의료기관 환경표면 소독 실무지침 수행도가 유의하게 높은 것으로 나타났으며, 현 근무부서 경력이 5년 이상인 간호사는 1~3년 미만 및 3~5년 미만인 간호사에 비해 수행도가 높은 것으로 확인되었다. 이러한 결과는 간호사의 근무 환경과 임상 경험이 의료기관 환경표면 소독 실무지침 수행과 밀접하게 관련되어 있음을 시사한다. 먼저, 상급종합병원 간호사의 수행도가 종합병원 간호사보다 높게 나타난 결과는 Jeong 등[20]이 상급종합병원 간호사가 종합병원 및 요양병원 간호사보다 환경 청소·소독 수행도가 높았다고 보고한 연구와 Kim [37]이 700병상 이상 대학병원 간호사의 표준주의 수행도가 300병상 미만 병원 간호사보다 높았다고 보고한 연구 결과와 일치한다. 이러한 일관된 결과는 의료기관의 규모 및 조직적 특성이 의료기관 환경표면 실무지침 수행 수준과 관련이 있을 가능성을 시사한다. 상급종합병원은 상대적으로 체계화된 감염관리 조직, 정기적인 교육 프로그램, 충분한 인력 및 자원 지원, 그리고 강화된 질 관리 체계를 갖추고 있을 가능성이 높다. 이러한 조직적 환경은 간호사의 감염관리 실무지침 수행을 촉진하는 구조적 기반으로 작용하여 환경표면 소독 실무지침 수행도에도 긍정적인 영향을 미쳤을 것으로 해석된다. 반면, 상대적으로 규모가 작은 의료기관에서는 인력 부족이나 업무 부담 증가, 교육 기회의 제한 등이 낮은 수행 수준과 관련이 있을 가능성도 있다. 다만 본 연구에서는 의료기관 규모에 따른 세부적인 조직 특성(예: 감염관리 인력 배치, 교육 빈도, 자원 지원 수준 등)에 따른 차이를 직접적으로 분석하지 못하였으므로, 향후 연구에서는 이러한 변수를 포함한 다층적 분석을 통해 수행도 차이에 영향을 미치는 기전을 보다 구체적으로 규명할 필요가 있다.

한편, 현 근무부서 경력이 길수록 의료기관 환경표면 소독 실무지침 수행도가 높게 나타난 결과는 근무 경력이 증가할수록 표준주의 수행도가 향상된다고 보고한 선행연구들[22,38,39]과 일치한다. 이는 임상경험의 축적을 통해 감염 노출 상황에 대한 인지와 대응 능력이 향상되고, 반복적인 교육 및 의료기관 인증평가 준비 과정 등을 통해 감염예방 및 관리의 중요성에 대한 인식이 강화되는 등 복합적인 요인이 작용한 결과로 해석될 수 있다. 그러나 본 연구에서는 경력과 관련된 교육 경험, 감염관리 인식 수준, 조직문화 등 잠재적 매개요인을 직접적으로 측정하지 못하였다는 제한점이 있다. 따라서 향후 연구에서는 근무경력과 함께 교육, 조직문화, 감염관리 체계 등 다양한 요인을 포함한 통합적 모형을 적용하여 수행도에 영향을 미치는 경로를 보다 체계적으로 분석할 필요가 있다. 본 연구에서 확인된 의료기관 규모 및 근무경력에 따른 수행도 차이는 의료기관 환경표면 소독 실무지침 수행이 개인의 인식이나 의지에만 의해 결정되기보다는 조직적 환경과 구조적 여건에 의해 영향을 받을 수 있음을 시사한다. 즉, 환경표면 소독 수행은 개인적 요인과 조직적 요인이 상호작용한 결과로 이해할 수 있으며, 수행도 향상을 위해서는 개인 수준의 교육과 더불어 조직 차원의 지원 체계 구축이 병행되어야 할 필요가 있다.

본 연구는 임상간호사의 의료기관 환경표면 소독 실무지침 수행에 영향을 미치는 요인을 개인적 요인과 조직적 요인의 관점에서 통합적으로 규명하고자 수행되었다. 개인적 요인으로는 의료기관 환경표면 소독 실무지침 중요도와 근거기반실무 신념을 포함하였으며, 조직적 요인으로는 근거기반실무 조직준비도를 고려하여 분석하였다. 의료기관 환경표면 소독 실무지침 수행도에 영향을 미치는 요인을 직접적으로 규명한 선행연구는 제한적이므로, 본 연구에서는 감염관리 수행 및 근거기반실무 수행 관련 선행연구와의 비교를 통해 결과를 해석하고자 하였다.

본 연구 결과, 의료기관 환경표면 소독 실무지침 수행도에는 의료기관 환경표면 소독 실무지침 중요도, 근거기반실무 신념, 그리고 근거기반실무 조직준비도가 모두 유의한 관련을 보였으며, 이들 변수를 포함한 회귀모형은 수행도의 57.6%를 설명하는 것으로 나타나 비교적 높은 설명력을 나타냈다. 이는 환경표면 소독 실무지침 수행이 단일 요인에 의해 결정되기보다는 개인적 요인과 조직적 요인이 상호작용한 결과로 이해될 수 있음을 시사한다.

본 연구결과를 감염관리 수행도에 영향을 미치는 요인을 분석한 선행연구와 비교해 보면, Hong과 Park [40]은 간호사의 감염관리 수행도가 의료기관 인증제에 대한 인식과 감염관리 중요도 인식에 의해 유의하게 설명되며, 해당 모형이 수행도의 42.6%를 설명한다고 보고하였다. 비록 측정된 수행 영역과 변수에 차이가 있어 직접적인 비교에는 제한이 있으나, 감염 관련 실무에서 중요성에 대한 인식이 수행도와 밀접하게 관련된다는 점에서 본 연구 결과와 유사한 맥락으로 해석할 수 있다. 특히 본 연구에서 의료기관 환경표면 소독 실무지침 중요도 점수는 4점 만점에 3.66점으로 수행도 점수보다 높게 나타났으며, 이는 간호사들이 해당 실무의 중요성을 충분히 인식하고 있음에도 실제 수행에는 일정 수준의 격차가 존재함을 시사한다. 이러한 결과는 수술실 환경표면 청소 수행을 조사한 연구[35]에서 중요성 인식에도 불구하고 수행 과정에서 절차 누락이나 부적절한 수행이 빈번하게 관찰되었다고 보고한 결과와 일치한다. 따라서 환경표면 소독 수행은 단순한 중요도 인식만으로 충분히 설명되기 어려우며, 실제 수행 과정에서는 시간적 제약, 업무 부담, 조직적 지원 등 다양한 요인이 복합적으로 작용할 가능성이 있다.

또한 코로나바이러스감염증-19 팬데믹을 거치면서 감염 예방에 대한 사회적 및 조직적 관심이 증가하고, 감염관리 교육 강화 및 의료기관 인증평가 준비 과정에서 환경표면 소독의 중요성이 지속적으로 강조되어 온 점 역시 수행도에 영향을 미치는 맥락적 요인으로 고려될 수 있다. 다만 이러한 해석은 본 연구에서 직접적으로 측정된 변수가 아니므로, 관련성 수준에서 신중하게 해석할 필요가 있다.

한편, Yoo 등[27]은 상급종합병원 간호사를 대상으로 한 연구에서 근거기반실무 수행도가 근거기반실무 신념, 조직준비도, 그리고 지식과 유의한 관련을 보였으며, 이들 변수가 수행도의 17.7%를 설명한다고 보고하였다. 본 연구에서도 환경표면 소독 실무지침 수행도가 근거기반실무 신념과 조직준비도와 유의한 관련을 보였다는 점에서, 감염관리 수행과 근거기반실무 수행 모두에서 개인의 신념과 조직적 준비도가 중요한 역할을 한다는 점을 확인할 수 있었다. 이러한 결과는 간호사의 실무지침 수행이 단순히 지식 수준에 의해 결정되기보다는, 실무지침의 중요성에 대한 인식, 근거기반실무에 대한 신념, 그리고 조직이 근거기반실무를 수행할 수 있도록 얼마나 준비되어 있는지와 같은 다차원적 요인과 관련됨을 의미한다. 따라서 효과적인 수행 향상을 위해서는 개인 수준의 교육뿐 아니라 조직 차원의 구조적 지원이 병행되어야 할 필요가 있다.

본 연구는 간호사의 개인적 요인뿐 아니라 조직적 요인인 근거기반실무 조직준비도가 의료기관 환경표면 소독 실무지침 수행과 유의한 관련이 있음을 확인하였다는 점에서 의의가 있다. 이는 개인의 인식과 태도 중심으로 수행도를 설명한 선행연구[25]를 확장하여, 개인적 요인과 조직적 요인을 통합적으로 고려할 필요성을 제시한 연구[32]의 관점을 지지하는 결과로 해석될 수 있다. 또한 Oh와 Park [41]의 연구에서 표준감염지침 수행의 저해 요인으로 인지 부족과 업무 과다가 보고된 점은, 개인 수준의 인식뿐 아니라 조직 차원의 업무 환경과 지원 체계가 수행에 중요한 영향을 미칠 수 있음을 뒷받침한다. 따라서 의료기관 환경표면 소독 실무지침 수행을 향상시키기 위해서는 간호사를 대상으로 한 실무지침 중심 교육과 더불어, 충분한 인력 확보, 업무 부담 완화, 표준화된 수행 절차 마련, 지속적인 모니터링 및 피드백 체계 구축 등 조직 차원의 준비가 함께 이루어져야 할 필요가 있다.

본 연구는 임상 현장에서 간호사의 환경표면 소독 수행 수준을 체계적으로 평가할 수 있는 도구를 개발하였다는 점에서 실무적 의의가 있다. 또한 의료기관 환경표면 소독 실무지침 중요도가 수행도의 가장 강력한 예측 요인으로 확인됨에 따라, 단순한 지식 전달 중심 교육보다 태도 및 인식 변화를 유도하는 교육 전략의 필요성을 제시하였다는 점에서 교육적 의의가 있다. 더불어 근거기반실무 신념과 조직준비도가 수행도와 유의한 관련을 보였다는 점은, 개인의 근거기반 신념을 강화하는 교육과 함께 조직 차원의 제도적 기반 구축이 병행되어야 함을 시사한다. 마지막으로 본 연구는 간호사의 개인적 요인과 조직적 요인을 통합적으로 분석하여 의료기관 환경표면 소독 실무지침 수행에 영향을 미치는 요인을 규명함으로써, 향후 간호 인력 지원, 물리적 자원 제공, 감염관리 모니터링 시스템 구축 등 조직 차원의 중재 연구를 위한 이론적·경험적 근거를 제시하였다는 점에서 연구적 의의가 있다.

그러나 본 연구는 비확률 표집 방법을 사용하였으며, 의료기관 규모별 표본이 균형 있게 확보되지 못하였고 일부 일반적 특성에서 소수의 표본이 포함되었다는 제한점이 있다. 또한 수행도를 객관적으로 측정하지 못하고 주관적 설문지로 측정하여 대상자의 특성에 따라 과대 또는 과소평가될 가능성이 있다는 제한점이 있다. 따라서 연구 결과의 일반화에는 신중한 해석이 요구된다. 향후 연구에서는 의료기관 규모 및 조직 특성을 고려한 체계적인 표본 설계를 적용하고, 다양한 조직적 요인을 포함한 확장된 분석을 통해 본 연구 결과를 검증할 필요가 있으며, 간호사들의 의료기관 환경표면 소독 수행도를 정확하게 평가하기 위해서는 관찰법을 이용하여 객관적으로 평가하는 것이 필요하다.

## 결론

본 연구에서 임상간호사의 의료기관 환경표면 소독 실무지침 수행에 의료기관 환경표면 소독 실무지침 중요도, 근거기반실무 조직준비도와 근거기반실무 신념이 유의하게 영향을 미치는 것으로 나타났다. 따라서 임상간호사의 의료기관 환경표면 소독 실무지침 수행도를 향상시키기 위해 간호사의 개인적 요인인 실무지침 중요도 인식과 근거기반실무 신념을 강화시킬 수 있는 교육이 필요하며, 조직적 측면으로는 근거기반실무 조직준비도를 강화시킬 수 있는 정책을 마련하는 것이 필요하다.

## Figures and Tables

**Table 1.** Differences in Disinfection Practice Performance by Participant Characteristics (N = 135)

Variables	Categories	n (%)	Mean ± SD	t/F (*p*) Scheffé
Age (years)	≤ 25	16 (11.9)	3.42 ± 0.40	2.89^✝^ (.299)
	26–30	76 (56.3)	3.24 ± 0.49	
	31–35	24 (17.8)	3.32 ± 0.46	
	≥ 36	19 (14.1)	3.43 ± 0.49	
Sex	Men	7 (5.2)	3.39 ± 0.48	0.47 (.638)
	Women	128 (94.8)	3.30 ± 0.48	
Education level	Associate’s degree (3-year)	8 (5.9)	3.38 ± 0.36	1.13 (.326)
	Bachelor’s degree (4-year)	93 (68.9)	3.26 ± 0.49	
	Graduate degree (≥ Master’s)	34 (25.2)	3.40 ± 0.47	
Length of clinical experience (years)	< 3	25 (18.5)	3.19 ± 0.57	4.14^✝^ (.218)
	3–< 5	22 (16.3)	3.18 ± 0.37	
	5–< 10	68 (50.4)	3.36 ± 0.46	
	≥ 10	20 (14.8)	3.39 ± 0.49	
Workplace	Tertiary hospital	72 (53.3)	3.40 ± 0.44	2.60 (.011)
	General hospital	63 (46.7)	3.19 ± 0.50	
Work unit	General ward	68 (50.4)	3.28 ± 0.49	−0.47 (.639)
	Specialized unit	67 (49.6)	3.32 ± 0.47	
Length of experience in the current department (years)	< 1^a^	30 (22.2)	3.31 ± 0.48	5.64 (.001) b,c < d
	1–< 3^b^	46 (34.1)	3.18 ± 0.50	
	3–< 5^c^	24 (17.8)	3.16 ± 0.46	
	≥ 5^d^	35 (25.9)	3.56 ± 0.36	
Experience of infection control education	Yes	125 (92.6)	3.31 ± 0.47	1.04 (.299)
	No	10 (7.4)	3.15 ± 0.63	

Post hoc analysis was conducted using the Scheffé test. SD = Standard deviation.

✝ Kruskal-Wallis test or Mann-Whitney U test.

**Table 2.** Descriptive Statistics of Study Variables (N = 135)

Item	n	Range	Cronbach's α	Mean ± SD
Performance of practical guidelines for disinfection of environmental surfaces	47	1-4	.96	3.27 ± 0.49
Types of detergents and disinfectants and selection criteria	4	1-4	.66	3.41 ± 0.48
Dilution concentration and preparation methods	2	1-4	.84	3.03 ± 0.84
Contact time	1	1-4	-	2.92 ± 0.99
Additional considerations	3	1-4	.62	3.30 ± 0.59
Equipment and supplies	5	1-4	.73	3.34 ± 0.54
Use of disinfectant wipes	3	1-4	.85	3.25 ± 0.71
General principles of disinfection	2	1-4	.79	3.22 ± 0.72
Frequency of disinfection	4	1-4	.83	3.34 ± 0.62
Direction of disinfection	7	1-4	.88	3.19 ± 0.66
Disinfection of high-touch surfaces	4	1-4	.83	3.31 ± 0.61
Surface disinfection for diarrhea-causing microorganisms	10	1-4	.89	3.36 ± 0.57
Terminal disinfection of patient rooms after discharge	2	1-4	.80	2.97 ± 0.95
Importance of practical guidelines for disinfection of environmental surfaces	47	1-4	.98	3.66 ± 0.35
Types of detergents and disinfectants and selection criteria	4	1-4	.83	3.60 ± 0.42
Dilution concentration and preparation methods	2	1-4	.87	3.61 ± 0.47
Contact time	1	1-4	-	3.57 ± 0.55
Additional considerations	3	1-4	.77	3.72 ± 0.38
Equipment and supplies	5	1-4	.86	3.65 ± 0.40
Use of disinfectant wipes	3	1-4	.86	3.60 ± 0.46
General principles	2	1-4	.82	3.67 ± 0.43
Frequency	4	1-4	.87	3.69 ± 0.40
Direction	7	1-4	.90	3.62 ± 0.44
High-touch surfaces	4	1-4	.87	3.70 ± 0.40
Diarrhea-causing microorganisms	10	1-4	.93	3.72 ± 0.37
Patient rooms after discharge	2	1-4	.82	3.57 ± 0.53
Evidence-based practice beliefs	16	1-5	.89	3.75 ± 0.48
Organizational readiness for evidence-based practice	25	1-5	.96	3.19 ± 0.78

Item (n) indicates number of questionnaire items. SD = Standard deviation.

**Table 3.** Relationships among Study Variables (N = 135)

Variables	Importance	Belief	Readiness
	r (*p*)		
Belief	.38 (< .001)	1	-
Readiness	.39 (< .001)	.54 (< .001)	1
Performance	.68 (< .001)	.50 (< .001)	.53 (< .001)

Values are Pearson correlation coefficients (r) with *p*-values in parentheses. Importance = Importance of practical guidelines for disinfection of environmental surfaces; Belief = Evidence-based practice beliefs; Readiness = Organizational readiness for evidence-based practice; Performance = Performance of practical guidelines for disinfection of environmental surfaces.

**Table 4.** Factors Influencing the Performance of Practical Guidelines for Disinfection of Environmental Surfaces (N = 135)

Variables	B	SE	β	t	*p*	95% CI		VIF
						Low	Upper	
(constant)	−0.52	.32		-1.65	.101	−1.15	0.10	
Importance of practical guidelines for disinfection of environmental surfaces	0.74	.09	.53	8.40	< .001	0.57	0.92	1.24
Evidence-based practice beliefs	0.18	.07	.18	2.55	.012	0.04	0.31	1.50
Organizational readiness for evidence-based practice	0.14	.04	.22	3.21	.002	0.05	0.22	1.50
R^2^	.58							
Adjusted R^2^	.57							
F (*p*)	59.31 (< .001)							

SE = Standard error; CI = Confidence interval; VIF = Variance inflation factor.
